# Appellation Preferences of Parents of Children Attending Hospital

**DOI:** 10.1177/00099228211072972

**Published:** 2022-01-28

**Authors:** Shahid Iqbal, Youssef Ibrahim, Massimo Garriboli

**Affiliations:** 1 GKT School of Medical Education, Faculty of Life Sciences & Medicine, King’s College London, London, UK; 2 Paediatric Urology, Evelina London Children’s Hospital, London, UK; 3 Stem Cells & Regenerative Medicine Section, Developmental Biology & Cancer Programme, UCL Institute of Child Health, London, UK

**Keywords:** communication, patient and family-centered care, parent satisfaction, pediatrics, interpersonal skills

## Abstract

Communication, carer-health care professional relationship, and power dynamics are important considerations in pediatric health care. There is paucity of evidence about best practice for addressing parents of children in a hospital care setting, potentially affecting health care provision. We surveyed parents attending Evelina London Children’s Hospital to assess the preferences of parents to different appellations used by health care professionals to address them and their impact on parents’ perception of involvement in the care of their child. Two hundred fifty-four (84.6%) parents responded to the survey. Two hundred one (92.6%) parents did not feel the way they were addressed contributed to them feeling their value was neglected from the care of their child. At the center studied, appellations most acceptable to parents were their first name or “Mum”/“Dad.” In current practice, the appellation used most is “Mum”/“Dad,” 112 (69.1%) and 40 (62%), respectively.

## Introduction

Positive perceptions of quality of care are predicted by specific clinical communication behaviors,^1^ and pediatric health care teams have the added responsibility of ensuring the care they provide is not just patient-oriented but also family-centred.^2^ Parents form a unique part of the team and their considerations and opinions have value in decision making. It is therefore essential to ensure they feel valued and an integral part of the team.^3^ Poor communication from physicians and other health care staff can impede the team-building process. It is also associated with an increased risk of malpractice claims^4-6^ and complaints to medical regulatory authorities.^7^ Thus, it is important for health care professionals to properly address parents of children attending hospitals. The relationship between the health care team and parents starts as soon as the initial encounter and greetings^8,9^ and develops as interactions increase. The “#hellomynameis” campaign^10^ that started in 2013 emphasized the importance of communication in caring for patients, largely by providing guidance for introducing the health care professionals to the patient and the appellation preferred by adult patients has been studied for a long time^11^; however, there is a paucity of literature on the preferred appellation of parents accompanying their child accessing health care services. One US study has showed that, when being greeted, most fathers preferred the title “Dad” (69.8%) and most mothers preferred the title “Mom” (79.8%).^12^ However, a recent report suggested there may be controversies and disapproval at being called “Mum” by a doctor, arguing that health care professionals should ask parents how they preferred to be addressed in order to empower them to be part of the clinical decision-making team.^13^ We aimed to explore the opinion of parents of children attending our tertiary referral hospital with regard to their preference of appellation by health care professionals, appellations used commonly by health care professionals to address parents, and whether undesirable appellation had an impact on parent perception of involvement in the care of their child.

## Methods

### Design

A cross-sectional survey study using an 11-item questionnaire developed with 2 gender-specific versions in English (see supplementary material, available online). Following ethical approval granted by Guy’s and St Thomas’ central QIPS team, quality and assurance directorate in February 2020, the survey was distributed by a medical student unrelated to the care of respective patients to English-speaking parents in inpatient and outpatient settings of a tertiary referral hospital: Evelina London Children’s Hospital (ELCH).

### Setting

ELCH is a 215-bed children’s specialist hospital located in central London with an admission rate of around 23 000 children per year. ELCH admits patients from birth to 18 years of age. All participants had children either admitted to one of the inpatient wards or had an outpatient appointment scheduled on the same day.

### Participants

Only parents who had an encounter with a health care professional on the same visit were included for participation; all other relatives or legal guardians were excluded. In inpatient wards and outpatient clinics, all potential participants who were available were approached and invited to take part in the survey. Eligibility to participate was confirmed prior to the distribution of the survey and verbal informed consent was given by participants.

### Questionnaire Design

Data collected included participant age, ethnicity,^14^ and education level (none, primary, secondary, or higher). Questions were asked to assess the perception of respondents to the condition of their child. Specifically, respondents were asked if they perceived their child to have a minor/major condition with an additional option for “don’t know.” Respondents’ perception of acuity/chronicity of their child’s condition was also assessed. Commonly used appellations were evaluated through asking respondents to choose the one appellation that had been used the most by health care professionals in their clinical encounter from: Mum/Dad, Mummy/Daddy or similar names of endearment, first name, last name, Ma’am/Sir, avoided use of a title/name, or other. Respondents were then asked if their preference of appellation was sought by health care professionals through a polar question. To determine the perceived importance of respondents to being asked how they prefer to be called by health care professionals, respondents were asked to evaluate a statement through a 5-level Likert-type scale (from 1 = “strongly disagree” to 5 = “strongly agree”). Respondents were then invited to evaluate their preference by scaling different appellations using a 5-level Likert-type scale from “strongly dislike” to “like a lot.” These appellations included the following: Mum/Dad, Mummy/Daddy, Ma’am/Sir, avoiding use of title or name, Miss/Mrs/Mr, and first name. Finally, respondents were asked to evaluate whether they felt the way they had been addressed neglected their value in the care of their child (see full questionnaire in supplementary material, available online).

### Data Analysis

Descriptive statistics were calculated for appropriate data. Associations among variable were assessed using cross-tabular analyses and the χ^2^ test of independence. Likert-type scale data were treated as ordinal (1-5) and subsequently analyzed using the Mann-Whitney *U* test when appropriate or the Kruskal-Wallis *H* test and post hoc Dunn’s test with *P* values adjusted according to the Benjamini-Hochberg FDR method. All significance was assessed at *P* = .05.

## Results

**Figure 1.**
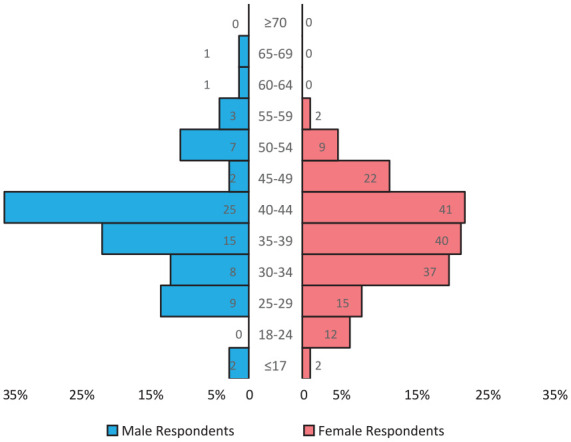
Age of respondents

The questionnaire was distributed to 300 parents. Forty-six (15%) refused to participate. Of those who refused, 1 refused because of stress, 14 refused because of lack of time, and 31 stated no or other reason for refusal. A total of 254 parents (85%) returned the questionnaire, of which 181 (71.2%) were mothers and 73 (28.7%) were fathers. Among the fathers there was 2 stepfathers (Table 1, Figure 1).

**Table 1.** Demographics of Respondents (N = 248)

Characteristics	n	Percentage
Parent gender		
Male	73	28.7
Female	181	71.3
Ethnicity		
White	169	66.5
British	139	
Gypsy/Traveler	1	
Irish	1	
Other	28	
Asian	17	6.7
Bangladeshi	2	
Pakistani	5	
Indian	7	
Other	3	
Black	41	16.1
African	27	
Caribbean	7	
Other	7	
Chinese	0	
Chinese	0	
Mixed	14	5.5
White/Asian	2	
White/Black African	2	
White/Black Caribbean	4	
Other mixed	6	
Other	7	2.8
Arab	5	
Any other	2	
Ethnicity not stated	6	2.4
Education level		
None	3	1.2
Primary education	4	1.6
Secondary education	96	37.8
Higher education	148	58.3
Not stated	3	1.2

**Figure 2.**
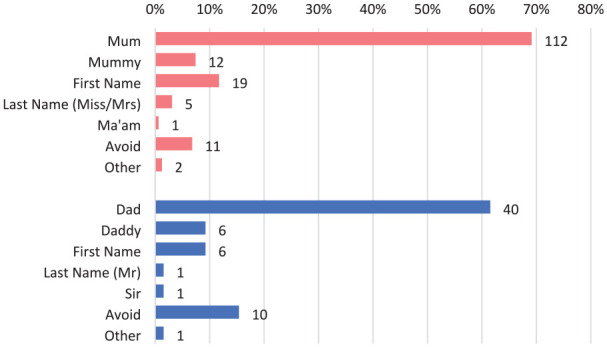
Commonly used appellations for mothers (red) and fathers (blue)

When mothers were asked which appellation had been used the most in their encounter with health care staff relating to the care of their child, 112 (69.1%) said “Mum,” 19 (11.7%) said it was their first name, 12 (7.4%) said “Mummy,” 11 (6.8%) said a title or name was avoided, 5 (3.1%) said it was their last name (Miss/Mrs), 2 (1.2%) said another appellation was used, and 1 (0.6%) said “Ma’am” (Figure 2).

When fathers were asked which appellation had been used the most in their encounter with health care staff relating to the care of their child, 40 (61.5%) said “Dad,” 10 (15.4%) said a title or name was avoided, 6 (9.2%) said “Daddy,” 6 (9.2%) said it was their first name, 1 (1.5%) said it was their last name (Mr), 1 (1.5%) said “Sir,” and 1 (1.5%) said another appellation had been used (Figure 2).

**Figure 3.**
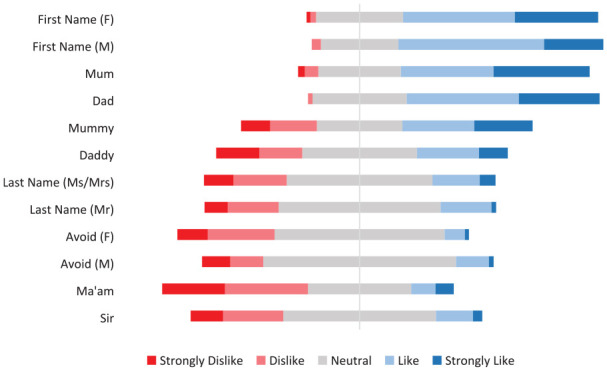
Parents’ perception of commonly used appellations. F, female respondent; M, male respondent

With regard to preference of different appellations for mothers, 59 (38.3%) “liked” and 44 (28.6%) “strongly liked” their first name being used. Fifty-five (31.8%) “liked” and 57 (32.9%) “strongly liked” the appellation “Mum.” Avoiding a title/name and the use of “Ma’am” were the least popular with 33 (22.9%) and 41 (28.5%), respectively, saying they “dislike” these, and 15 (10.4%) and 31 (21.5%) stating they “strongly dislike” these, respectively (Figure 2). With regard to preferences of different appellations for fathers, 32 (50.0%) “liked” and 13 (20.3%) “strongly liked” being called by their first name. Twenty-five (38.5%) “liked” and 18 (27.7%) “strongly liked” the appellation “Dad.” “Sir” was the least popular appellation with 13 (20.6%) stating the “dislike” this appellation and 7 (11.1%) stating they “strongly dislike” it. Overall, first name was the appellation liked the most by mothers and fathers (66.9% of mothers “like” or “strongly like” and 70.3% of fathers “like” or “strongly like”; Figure 3).

Eighty-three (33.5%) parents stated they were asked by health care professionals how they would like to be called, of which 43 (51.8%) agreed or strongly agreed it was important to them that health care professionals did so. Of the 165 (66.5%) parents who were not asked their appellation preference, 84 (51%) stated being neutral about the importance they held to being asked appellation preference by health care professionals.

**Figure 4.**
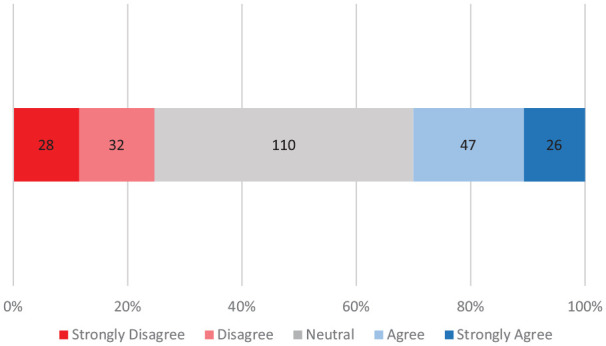
Parents’ perceived importance of being asked preferred appellation by health care providers

Parents’ opinion of a statement affirming the importance of health care staff asking them how they would like to be addressed differed greatly with almost half of responses being “neutral” (Figure 4). There was no significant difference between mothers’ opinion (mean Likert score: 3.06 ± 1.10) and fathers’ opinion (mean Likert score: 3.00 ± 1.11) of the importance of being asked how they would like to be called (*U* = 5813, *z* = 0.587, *P* = .5552). Overall, 70.0% (170) of parents did not feel being asked about preferences for appellations before interacting with health care professionals was important to them.

There was no significant difference in perceived importance of parents being asked how they would like to be addressed between self-reported acute (mean Likert score: 2.84 ± 1.18), chronic (mean Likert score: 3.15 ± 1.06), and unknown disease status (mean Likert score: 3.06 ± 1.08; *H*[2, n = 235] = 3.49, *P* = .18). However, there was a significant difference in perceived importance of parents being asked how they would like to be addressed between self-reported minor (mean Likert scale: 2.87 ± 1.12), major (mean Likert scale: 3.28 ± 1.09), and unknown (mean Likert scale: 2.96 ± 1.01) disease status (*H*[2, n = 239] = 7.468, *P* = .024). In post hoc analysis, there was a significant difference in the distribution of answers between major and minor disease status (*P* = .020).

In answering about opinions of different appellations, fathers chose a neutral response (45%) significantly more than mothers (38%; *z* = 2.37, *P* = .018).

White mothers displayed significantly higher disapproval at being addressed with the appellation “Ma’am” compared with their non-White counterparts (mean Likert scale: 2.30 ± 1.08 vs 2.98 ± 1.01; *U* = 1361.5, *z* = −3.50271, *P* = .00046).

Similarly, White mothers displayed significantly higher disapproval at being called with their last name (Miss/Mrs) compared with non-White mothers (mean Likert scale: 2.75 ± 1.06 against 3.20 ± 0.69; *U* = 1653.5, *z* = −2.54715, *P* = .01078).

There was no correlation between education level of parents and preference of different appellations.

Only 12 (7.8%) mothers stated they “agree” or “strongly agree” that the way they were addressed neglected their value in the care of their child. Of those, 9 (75%) stated they were addressed as “Mum” the most during their encounter with health care staff, 2 (16.7%) stated they were addressed the most by their first name and 1 (8.3%) stated a title or name was avoided.

Only 4 (6.4%) fathers reported they “agree” or “strongly agree” that the way they were addressed neglected their value in the care of their child. Of those, 2 (50%) stated they were most commonly addressed as “Dad” and 2 (50%) stated they were most commonly addressed by their first name.

## Discussion

To our knowledge, this is the first study performed in the United Kingdom that has evaluated parents’ preference of appellation used by health care professionals to address them or the impact of undesirable appellation. We explored commonly used parent appellations, preference of parents toward different appellations, perceived importance of parents being asked about appellation preference, and finally, whether undesirable appellation had an impact on parent perception of involvement in the care of their child.

Our results suggest that health care professionals should use either parents’ first name or the generic appellation of “Mum”/“Dad” while avoiding other appellations. There is little literature against which we can compare our results, though our findings are in line with a similar study published in the United States in 2018.^12^ These results suggest that the preference adult patients have for informal over formal address^11^ extends into parents accompanying their children to seek care.

Our sample was representative of a diverse London population, indeed, there were fewer White parents (66.5% here vs 86.0% nationally^15^) allowing us to make important comparisons across ethnic groups.^16,17^ It is interesting to note that White mothers seemed to dislike being addressed either as “Ma’am” or by their last name (Ms/Mrs) compared with their non-White counterparts. This finding is similar to what was reported in adult patients.^16^

Our sample contained fewer fathers than mothers, which reflects normal findings in pediatric health care settings.^8,9,12^ Nevertheless, we were still able to elicit views from 78 fathers, which represents a larger group compared with published literature.^12^ In giving their opinion of different appellations, fathers chose a neutral response significantly more than mothers, indicating that mothers hold more importance to the appellation used to address them. Most parents are currently referred to as “Mum”/“Dad.” Only a small minority of parents reported that the way they had been addressed undervalued their contribution to their child’s care. This suggests that although some appellations are unpopular, their use does not remove parents’ perception of their involvement in the team delivering health care to the child. It is worth noting the difference in perceived importance of being asked appellation preference between parents who were asked how they prefer to be called and those who were not. Those who were not asked their appellation preference perceived this to be relatively unimportant compared with those who were asked their appellation preference. This suggests being asked appellation preference by health care professionals could have an effect on the perception of its consequent importance by parents.

However, when the child’s condition was perceived as more serious, parents’ opinion shifted toward agreeing with the view that it was important for health care professionals to ask them how they wanted to be addressed. A serious condition could indicate higher levels of stress and a greater need for involvement in the care of the child, in which case parents also deem the appellation used to address them as important.

We were unable to elicit views from non-English-speaking parents, and thus our findings are only relevant to English-speaking parents. Additionally, our study being a single-center study limited our ability to sample a varied population thus limiting our ability to compare appellation preferences across cultures. For the sake of brevity and ease of comprehension, a nonstandard parent education scale^18^ was used, which may have affected the data collected but allowed us to capture data without the need for clarification of definitions. Our questionnaire did not allow for open-ended questions to understand potential reasons for respondents’ preferences, nor were we able to establish the family structure of respondents, or whether mothers had different last names to their child, which has previously caused physicians to use an undesirable appellation.^8^ Moreover, it is important to consider that it is not only parents who present with pediatric patients to hospital but also legal guardians or other relatives can often be seen involved with the care of children. Unfortunately, our study did not explore appellations and their impact in these populations, but this could be an important avenue for further research.

Further research should also explore the opinions and preferences of children about the way their parents are addressed when attending hospital and qualitatively explore how appellation preference might affect the perceived value of parents in the care of their child.

## Conclusion

A key role in the success of medical treatment relies in the relationship established between the health care professional and the patient. In pediatric settings, health care teams have the responsibility of providing patient and family-centered care. Poor communication can impede the team-building process between the health care team and parents of pediatric patients. With regard to the limitations of our study, our results suggest that the vast majority of parents do not feel that being asked their appellation preference was important, nor do they feel the way they were addressed neglected their value in the care of their child. Nevertheless, our results suggest that health care professionals should use informal appellation to address parents with either parents’ first name or the generic appellation of “Mum”/“Dad.”

## Author Contributions

SI: Contributed to conception and design; contributed to acquisition, analysis, and interpretation; drafted manuscript; critically revised manuscript; gave final approval; agrees to be accountable for all aspects of work ensuring integrity and accuracy.

YI: Contributed to design; contributed to acquisition, analysis, and interpretation; critically revised manuscript; gave final approval; agrees to be accountable for all aspects of work ensuring integrity and accuracy.

MG: Contributed to design; contributed to acquisition, analysis, and interpretation; critically revised manuscript; gave final approval; agrees to be accountable for all aspects of work ensuring integrity and accuracy.

## Supplemental Material

sj-pdf-1-cpj-10.1177_00099228211072972 – Supplemental material for Appellation Preferences of Parents of Children Attending Hospital Click here for additional data file. Supplemental material, sj-pdf-1-cpj-10.1177_00099228211072972 for Appellation Preferences of Parents of Children Attending Hospital by Shahid Iqbal, Youssef Ibrahim and Massimo Garriboli in Clinical Pediatrics

sj-pdf-2-cpj-10.1177_00099228211072972 – Supplemental material for Appellation Preferences of Parents of Children Attending Hospital Click here for additional data file. Supplemental material, sj-pdf-2-cpj-10.1177_00099228211072972 for Appellation Preferences of Parents of Children Attending Hospital by Shahid Iqbal, Youssef Ibrahim and Massimo Garriboli in Clinical Pediatrics
