# Supplementary material for: Appellation Preferences of Parents of Children Attending Hospital
Source: Clin Pediatr (Phila). 2022 Jan 28;61(3):289–94. doi: 10.1177/00099228211072972 (PMC8892054; doi:10.1177/00099228211072972)
Supplement: sj-pdf-2-cpj-10.1177_00099228211072972 – Supplemental material for Appellation Preferences of Parents of Children Attending Hospital [file sj-pdf-2-cpj-10.1177_00099228211072972.pdf]

For Office Use Only:
Ward/Department:
Inpatient/Outpatient/Day Surgery
Date:
Time:

n°

Attitudes & preferences of parents to the greeting used by healthcare professionals during the care of their child (M)

This questionnaire is **anonymised** and consists of 11 questions – Please turn over

Questionnaire

1) How are you related to the patient?

☐ Mother ☐ Stepmother

2) How old are you? **Please tick ONE**

☐ ≤ 17	☐ 18 - 24	☐ 25 - 29	☐ 30 - 34
☐ 35 - 39	☐ 40 - 44	☐ 45 - 49	☐ 50 - 54
☐ 55 - 59	☐ 60 - 64	☐ 65 - 69	☐ ≥ 70

3) What is your ethnic group? **Please tick ONE**

White
☐ White British
☐ White Gypsy/Traveller
☐ White Irish
☐ White Other: ……………

Asian
☐ Bangladeshi
☐ Pakistani
☐ Indian
☐ Asian Other: ……………

Black
☐ Black African
☐ Black Caribbean
☐ Black Other: ……………

Mixed
☐ Mixed White/Asian
☐ Mixed White/Black African
☐ Mixed White/Black Caribbean
☐ Any Other Mixed

Other
☐ Arab
☐ Any Other: ……………

Chinese
☐ Chinese

4) What is the **highest** level of education you have achieved? **Please tick ONE**

☐ None
☐ Primary Education (primary school)
☐ Secondary Education (GCSEs/A-levels)
☐ Higher education (university degrees, HE diplomas etc.)

5) My child's health condition is:

☐ Minor ☐ Major ☐ Don't know

6) My child's health condition is:

☐ Acute (short term) ☐ Chronic (long term) ☐ Don't know

7) Have you been asked by any of the healthcare staff how you would like to be called?

☐ Yes ☐ No

8) <u>"I feel it is important for healthcare professionals to ask me how I would like to be called before talking to me about my child"</u>

O________________O________________O________________O________________O

Strongly Disagree | Disagree | Neutral | Agree | Strongly agree

9) <u>What is the **ONE** greeting healthcare staff have used the most when talking to you directly during the care of your child?</u> **Please tick ONE**

☐ "Mum"

☐ "Mummy" or other like name of endearment, specify: ………………..

☐ Your first name

☐ Your last name i.e. Mrs/Miss

☐ Ma'am

☐ Avoided the use of a title or name

☐ Other, specify: ………………..

10) <u>How do you feel about the following greetings if they were used by the healthcare team when talking to you?</u>

a) "Mum"

O________________O________________O________________O________________O

Strongly Dislike | Dislike | Neutral | Like | Like a lot

b) "Mummy"

O________________O________________O________________O________________O

Strongly Dislike | Dislike | Neutral | Like | Like a lot

c) "Ma'am"

O________________O________________O________________O________________O

Strongly Dislike | Dislike | Neutral | Like | Like a lot

d) Avoiding use of any title or name

O________________O________________O________________O________________O

Strongly Dislike | Dislike | Neutral | Like | Like a lot

e) "Miss/Mrs"

O________________O________________O________________O________________O

Strongly Dislike | Dislike | Neutral | Like | Like a lot

f) First name

O________________O________________O________________O________________O

Strongly Dislike | Dislike | Neutral | Like | Like a lot

11) <u>Do you feel the way you have been called neglects your value in the care of your child?</u>

O________________O________________O________________O________________O

Strongly Disagree | Disagree | Neutral | Agree | Strongly agree
